# Research trends and hotspots of high tibial osteotomy in two decades (from 2001 to 2020): a bibliometric analysis

**DOI:** 10.1186/s13018-020-01991-1

**Published:** 2020-11-09

**Authors:** Haitao Zhang, Yinuo Fan, Rui Wang, Wenjun Feng, Jinlun Chen, Peng Deng, Xinyu Qi, Pengcheng Ye, Yijin Li, Jiahao Li, Jianchun Zeng, Yirong Zeng

**Affiliations:** 1 The First Clinical Medical School, Guangzhou University of Chinese Medicine, Jichang Road 12#, District Baiyun, Guangzhou, Guangdong China; 2 Department of Orthopaedics, The First Affiliated Hospital of Guangzhou University of Chinese Medicine, Jichang Road 16#, District Baiyun, Guangzhou, 510405 Guangdong China

**Keywords:** Bibliometric, Research trends, Hotspots, High tibial osteotomy

## Abstract

**Background:**

The purpose of this study is to comprehensively analyze the global application trend of high tibial osteotomy (HTO) and identify promising research hotspots of HTO based on bibliometrics and visual analysis.

**Methods:**

Publications (articles and reviews) related to HTO from 2001 to 2020 were retrieved from the Web of Science Core Collection database (WOSCC). The country, institution, year, author, journal, average citations per item, H index, title, abstract, keywords of publication, and the top 10 cited articles were extracted and analyzed in detail. The VOSviewer software was used to analyze theco-occurrence of keywords to predict the hotspots of HTO.

**Results:**

A total of 1883 articles were included. In the past 20 years, the number of HTO articles has shown an increasing trend in general. The top 3 countries (the USA, Germany, and South Korea) accounted for 49.547% of all articles published. The USA has the largest number of publications. The University of Western Ontario is the largest contributor. The *Knee Surgery Sports Traumatology Arthroscopy* is the most influential journal. Professors Saito T and Imhoff AB are the leading scholars who made great achievements in the HTO field. The research direction can be divided into the following 5 clusters: "prognosis and outcome", "HTO combined with cartilage restoration techniques", "animal experimental research", "study on bone union and plate fixation at osteotomy", and "surgical technique research".

**Conclusion:**

In terms of the trend of previous years, an increasing number of literatures related to HTO will be published in the future. The USA is a world leader in the field of HTO. South Korea presented great potential in this area. HTO combined with cartilage restoration techniques, postoperative prognosis and outcome, and surgical technique research may be the future hotspots in the field of HTO research.

## Introduction

Knee osteoarthritis (KOA) is a degenerative bone and joint disease that seriously affects the life quality of patients [1, 2]. Currently, the mainstream surgical approach for the treatment of knee osteoarthritis includes total knee arthroplasty (TKA), unicompartmental knee arthroplasty (UKA), and high tibial osteotomy (HTO). Although TKA and UKA have received a positive evaluation in elderly patients, HTO appears to be a more sensible option for many younger patients with high functional activity needs [3, 4]. As a mature method of “knee preservation,” HTO has been widely used in the treatment of unicompartmental knee osteoarthritis. The operation principle of HTO is to transfer the pressure load in unicompartment by adjusting the force line of the lower extremities so as to relieve pain, restore knee joint function, and prolong knee joint life [5]. Numerous studies have shown that HTO has been proven to be a reliable treatment with good short- and medium-term efficacy for KOA after years of clinical practice [6–8]. In recent years, a growing number of orthopedic surgeons have shown great interest in HTO surgery due to a substantial increase in the number of patients with KOA [9].

However, to the best of our knowledge, the recent research status and future research hotspots of HTO have not been well studied. In the context of the high profile of HTO, it is particularly meaningful to understand the global status quo of the field and predict future research hotspots. Bibliometrics is a multi-system discipline that integrates mathematics, statistics, philology, and other disciplines. Its advantage lies in the qualitative and quantitative evaluation of specific research areas based on multi-disciplines [10]. At present, bibliometrics has been extensively applied on numerous medical fields as a burgeoning method [11]. This study systematically analyzed the relevant literatures of HTO from 2001 to 2020 by using the Web of Science (WOS) database and VOSview software to provide a more comprehensive understanding of the global application trend and identify promising research direction in the future of HTO.

## Methods

### Data source and search strategies

The Web of Science Core Collection (WOSCC) is recognized as the most suitable online database for bibliometric analysis. We select the "Advanced search" function in the WOSCC online database and enter keywords to obtain the relevant literature of HTO from January 2001 to May 30, 2020 (the last 20 years). Document retrieval and export should be completed within 1 day to avoid bias caused by continuous database update. The retrieval strategy of this study was as follows: (Ts = high tibial osteotomy), refined by, document type (article OR review), and language (English).

### Data collection

All literature retrieval and data extraction were completed independently by two authors (Zhang and Fan) with familiar literature retrieval background. When they disagree, the differences are fully discussed until they reach an agreement. The data is saved in the TXT document format by using the built-in "create citation report" and "analyze search results" functions in WOSCC and in turn is imported into Microsoft Excel 2019 for further collation. The data include publishing country or region, institution, year, author, source journal, average citation times, H index, title, abstract, and keyword. The top 10 most-cited literatures were collected and preliminarily reviewed and analyzed. The relevant information of the top 10 most-cited literatures includes the first author, topic, citations, journal, and journal impact factor (IF). IF is obtained from the 2018 version of Journal Citation Reports (JCR).

### Bibliometric and visualized analysis

The world distribution map of HTO-related research and the forecast map of quantity growth trend of HTO publications are produced by using Microsoft Excel 2019. The citation frequency of the literature reflects the degree to which the article is recognized by scholars. H index is a qualitative and quantitative comprehensive index reflecting the academic level of scholars. This means that the citation frequency and the H index of the literature can also be extended to assess the academic contribution of a country, institution, journal, or author. Therefore, Microsoft Excel 2019 is used to draw a combination chart of the total number, H index, and average citations per item of publications, according to the grouping of countries, institutions, journals, and authors. VOSviewer (Van Eck and Walt-man, Leiden University, Leiden, The Netherlands) is a universally used visual tool supported by Java [12]. The downloaded TXT file is imported into VOSviewer1.6.15 for literature co-citation analysis and keyword co-occurrence analysis. In the visualization analysis figure, the larger the label, the more important the label, and the thicker the line between nodes indicate the closer relationship between the two nodes connected by the line.

## Results

### Analysis of publication trend in recent two decades

**Fig. 1:**
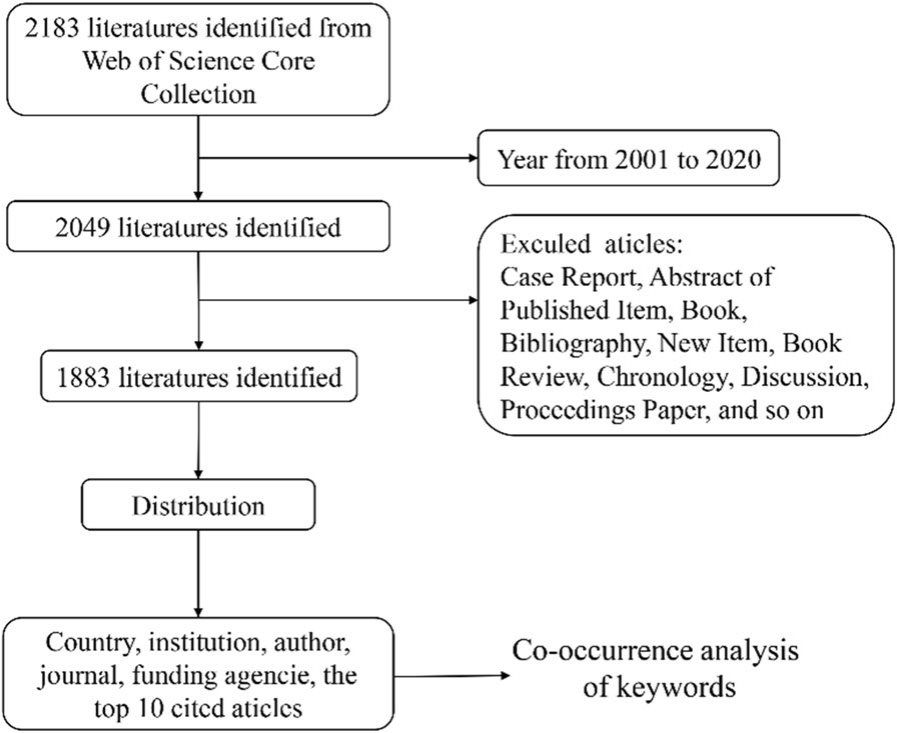
Flow chart of literature filtering included in this study

**Fig. 2:**
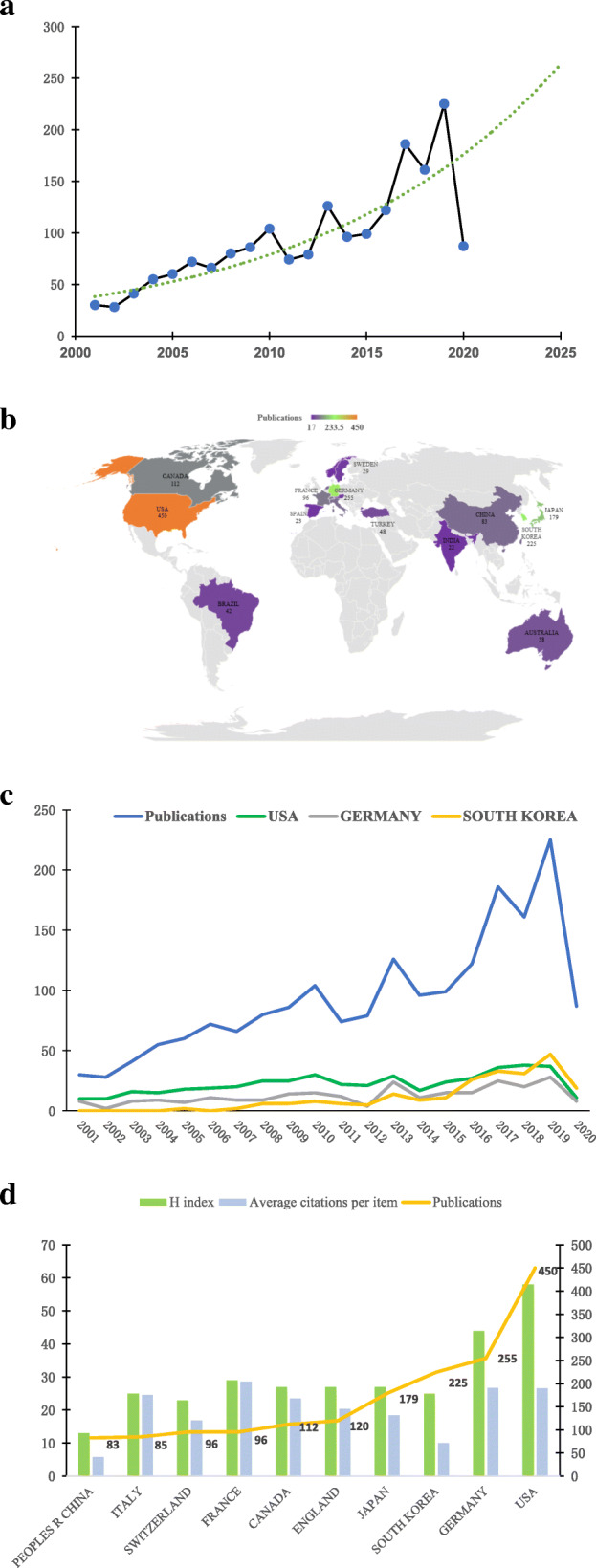
**a** Curves: growth trends of accumulated number of publications on HTO worldwide from 2001 to 2020. **b** Distribution of HTO research in the world map. **c** The number of publications from the USA, Germany, and South Korea over a 20-year period. **d** The total number of articles published, the H index, and the average citations per item in the top 10 countries

A total of 2049 publications related to HTO were searched, and 1883 met the criteria from 2001 to 2020 (Fig. 1). Figure 2a shows that although the number of publications related to HTO has fluctuated slightly over the past 20 years, it has presented an overall growth trend. The year 2019 (225 papers) is the peak of the number of literatures. It is predicted that the number of publications will reach 250 by 2025 according to the curve model.

### Distribution and contribution of the country (region)

Overall, the included literatures come from 63 different countries or regions (Fig. 2b). Figure 2c shows the number of publications from the USA, Germany, and South Korea over a 20-year period. The USA (450, 23.974%) is the country with the largest number of articles in the field of HTO research. Germany ranked second (255, 13.586%). In third place is South Korea (225, 11.987%). The top three countries accounted for 49.547% of all articles (Fig. 2d).

In addition, the USA ranked first in the H Index (58), followed by Germany (44), while France (27) ranked third. Surprisingly, France overtook the USA in the average citations per item, while the USA (26.58) and Germany (26.77) ranked second and third with almost the same number.

### Highly contributive institutions on HTO

**Fig. 3:**
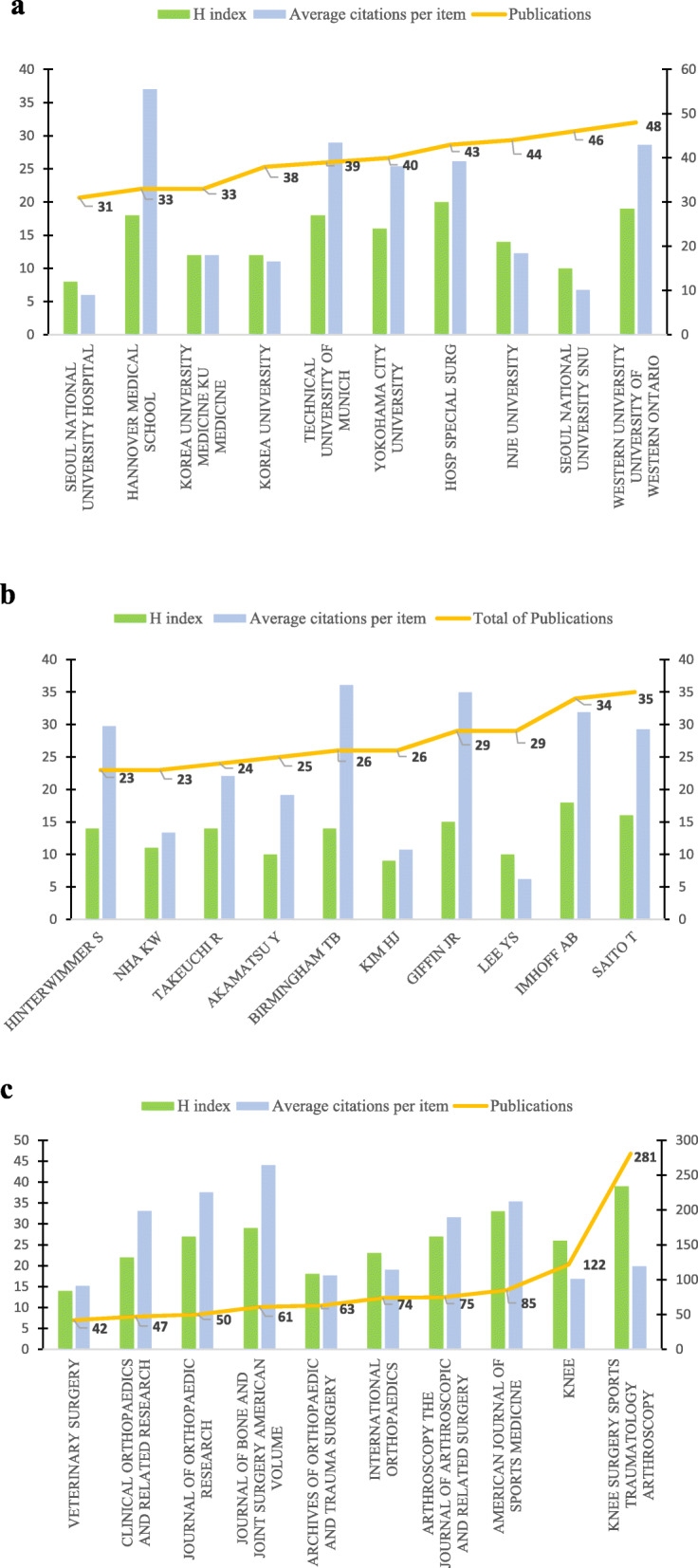
A combination chart of the total publications, H index, and average citations per item. **a** About institutions. **b** About authors. **c** About journals

Figure 3a shows the 10 organizations that have contributed the most in the field of HTO. Among them, the University of Western Ontario from Canada published the most articles (48 papers). This was followed by Seoul National University SNU (46 papers) and Inje University (44 papers) from South Korea. However, the top three institutions of the H index are HOSP SPECIAL SURG (USA, 20), University of Western Ontario (USA, 19), and Hannover Medical School (Germany, 18). Regarding the average citation frequency, the Hannover Medical School (37) ranked first, followed by the Technical University of Munich (Germany, 28.95) and the University of Western Ontario (28.63).

### Highly contributive authors on HTO

Saito T (35, H index = 16) and Imhoff AB (35, H index = 18) rank in the top two with almost equal number of posts and H index. Although Birmingham TB’s articles are cited more frequently (36.08), the number (25) is much lower than the previous two authors (Fig. 3b).

### Highly contributive journals on HTO

The *Knee Surgery Sports Traumatology Arthroscopy* has published the most HTO surgery papers (IF = 3.149, 281), followed by the *Knee* (1.762, 122) and the *American Journal of Sports Medicine* (6.093, 85). *Knee Surgery Sports Traumatology Arthroscopy* (39) has the highest H index. However, the *Journal of Bone and Joint Surgery American Volume* (29) and *Arthroscopy: the Journal of Arthroscopic and Related Surgery* (27) which publicated smaller number papers have higher H index. A similar situation also appears in the average citation frequency (Fig. 3c).

### Distribution of highly contributive funding agencies

The top 10 funding organizations are shown in Table 1, including 3 from the USA, 4 from South Korea, and 2 from Germany.

**Table 1:** The top 10 related funding agencies

Funding agency	Number	%
National Institutes of Health (NIH) USA	37	1.971
United States Department of Health Human Services	37	1.971
Canadian Institutes of Health Research (CIHR)	25	1.332
Arthrex	23	1.225
NIH National Institute of Arthritis Musculoskeletal Skin Diseases (NIAMS)	20	1.066
National Natural Science Foundation of China	17	0.906
Smith Nephew	16	0.852
German Research Foundation (DFG)	14	0.746
Canada Research Chairs	13	0.693
Arthrex Inc.	12	0.639

### Characteristics of the top 10 HTO articles

As for the top 10 articles with total citation frequency, the study by Zhang et al., published in the *Osteoarthritis and Cartilage* in 2008, had the most total citation frequency (1569). Its total citation frequency is far higher than that of other articles. Of the ten articles, three were published in *Osteoarthritis and Cartilage* and two in *Knee Surgery Sports Traumatology Arthroscopy* (Table 2).

**Table 2:** The top 10 HTO research papers with the most citation frequency

Rank	Topic	First author	Year	Journal	Impact factor	Citations	Major conclusion
1	OARSI recommendations for the management of hip and knee osteoarthritis, part II: OARSI evidence-based, expert consensus guidelines	Zhang, W.	2008	*Osteoarthritis and Cartilage*	4.879	1569	They developed 25 expert consensus recommendations for the treatment of osteoarthritis of the hip and knee (OA)
2	Human autologous culture expanded bone marrow mesenchymal cell transplantation for repair of cartilage defects in osteoarthritic knees	Wakitani, S	2002	*Osteoarthritis and Cartilage*	4.879	631	This procedure highlights the availability of autologous culture expanded bone marrow mesenchymal cell transplantation for the repair of articular cartilage defects in humans
3	Improvements in surgical technique of valgus high tibial osteotomy	Lobenhoffer, P	2003	*Knee Surgery Sports Traumatology Arthroscopy*	3.149	270	They present four technical modifications of high tibial osteotomy which improve its safety and reproducibility
4	Treatment of anterior cruciate ligament injuries, part I	Beynnon, BD	2005	*American Journal of Sports Medicine*	6.093	260	They review the treatment of anterior cruciate ligament injuries
5	Joint injury causes knee osteoarthritis in young adults	Roos, EM	2005	*Current Opinion in Rheumatology*	3.851	249	Knee injury prevention, rehabilitation after knee injuries, regular exercise, maintaining body weight, and a changed locomotion pattern may prevent osteoarthritis initiation and progression in young adults
6	Control of frontal plane knee laxity during gait in patients with medial compartment knee osteoarthritis	Lewek, MD	2004	*Osteoarthritis and Cartilage*	4.879	219	The presence of medial laxity in patients with knee OA is likely contributing to the altered gait patterns observed in those with medial knee OA
7	TomoFix: a new LCP-concept for open wedge osteotomy of the medial proximal tibia—early results in 92 cases	Staubli, AE	2003	*Injury-International Journal of the Case of the Injured*	1.834	214	A new fixation device (TomoFix (TM)) with an adapted surgical technique allows stable fixation of the osteotomy without the need to fill the osteotomy gap with bone grafts
8	Meniscal allograft transplantation: long-term clinical results with radiological and magnetic resonance imaging correlations	Verdonk, Peter C. M.	2006	*Knee Surgery Sports Traumatology Arthroscopy*	3.149	212	Long-term results after viable meniscus allograft transplantation are encouraging in terms of pain relief and improvement of function
9	Tibial osteotomy for the treatment of varus gonarthrosis—survival and failure analysis to twenty-two years	Sprenger, TR	2003	*Journal of Bone and Joint Surgery American Volume*	4.716	206	There is a role for tibial osteotomy, as an alternative to total knee arthroplasty, in patients who are less than 60 years old
10	Decreased knee adduction moment does not guarantee decreased medial contact force during gait	Walter, Jonathan P.	2010	*Journal of Orthopaedic Research*	3.043	199	Future studies that evaluate the effectiveness of gait modifications for offloading the medial compartment of the knee should consider the combined effect of these two knee moments

### Hotspots analysis of HTO surgery

**Fig. 4:**
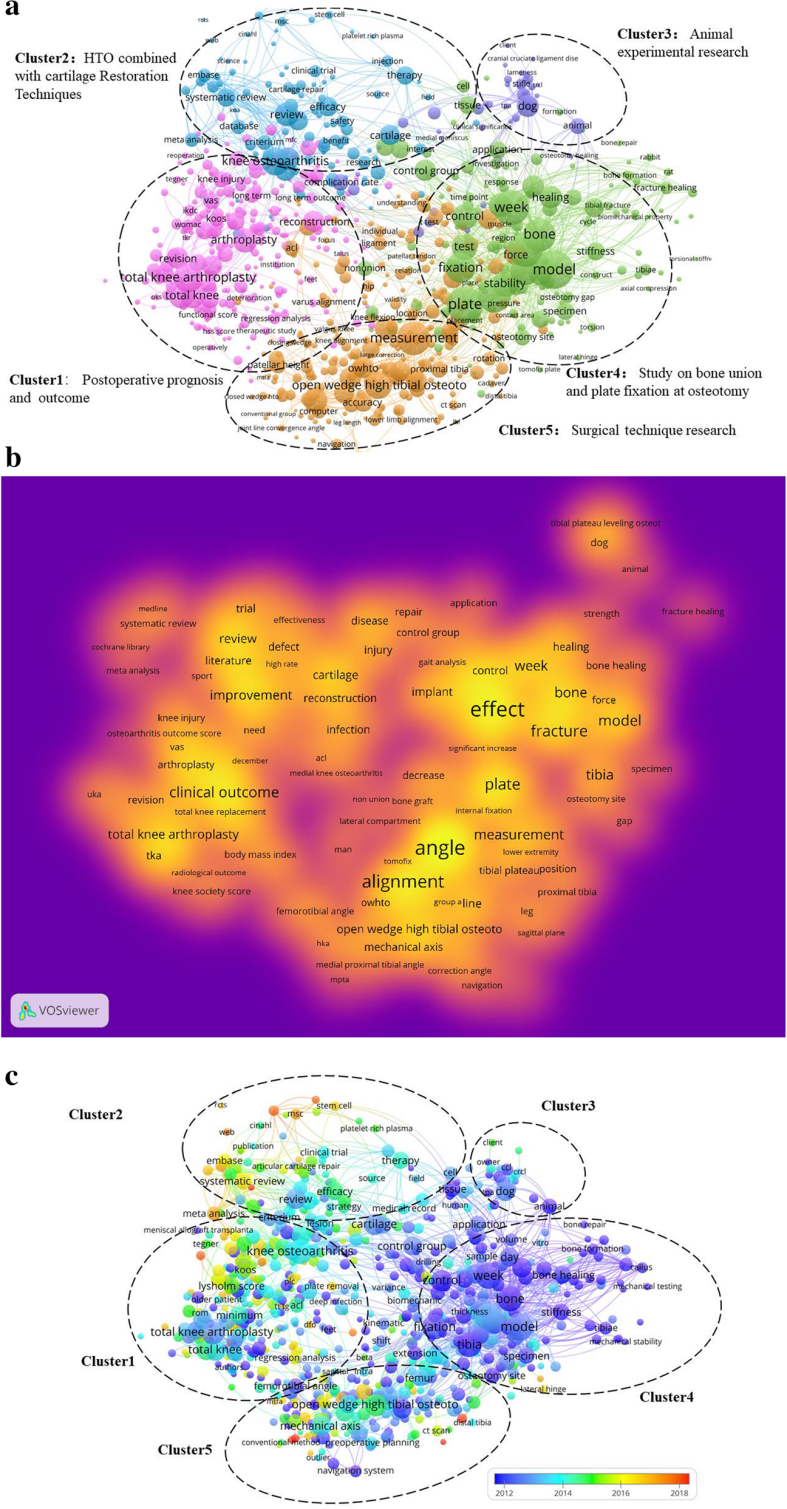
Co-occurrence analysis of HTO. **a** Mapping of keywords in the research area. The size of the points represents the frequency of appearance, and the keywords are divided into five clusters. **b** Heat map of keywords according to the mean frequency of appearance. **c** Overlap visualization diagram of color variation with the time axis (2012–2018); blue and purple nodes appear later than yellow and red nodes, meaning that red and yellow nodes are hot keywords

The co-occurrence analysis of keywords is a significant method to identify the hotspots of HTO research. The principle is to identify the importance of keywords by calculating the number of times that keywords appear repeatedly in all the titles and abstracts of included literatures. Setting the counting method to binary counting, the minimum number of recurring keywords is 10. The 692 keywords that reached the threshold are shown in Fig. 4a and are divided into five clusters. These five categories represent the main research directions in the field of HTO during the past 20 years. The five clusters are “HTO postoperative prognosis and outcome” (cluster 1, purple), “HTO combined with cartilage restoration techniques” (cluster 2, light blue), “Animal experimental research” (cluster 3, dark blue), “Study on bone union and plate fixation at osteotomy” (cluster 4, green), and “Surgical technique research” (cluster 5, yellow) (Fig. 4 a, c). In cluster 1, the most striking keywords are total knee arthroplasty, WOMAC, reconstruction, long-term outcome, KOOS, VAS, and functional score. In cluster 2, the more common keywords are knee osteoarthritis, cartilage, efficacy, and stem cell. In cluster 3, the keywords with the most repetition are animal, rat, rabbit, dog, and stifle. In cluster 4, the most popular keywords are fixation, stability, plate, bone, healing, and osteotomy gap. In cluster 5, the most prominent keywords are open wedge high tibial osteotomy, proximal tibial, closed wedge, joint line convergence angle, and knee alignment.

An overlap visual rainbow diagram (Fig. 4 b) is drawn based on the approximate year in which the keyword appears, and the color varies with the timeline (2012–2018). Blue and purple nodes appear later than yellow and red nodes, meaning that red and yellow nodes are hot keywords. As can be seen from the figure, although most of the studies before 2014 focused on cluster 3 and cluster 4, after 2016, most of the hot keywords concentrated on cluster 1, cluster 2, and cluster 5.

## Discussion

In this study, we systematically studied the HTO surgery nearly 20 years of the present situation and development trend by combining with the bibliometrics and visualization analysis. We analyzed countries, institutions, journals, and authors that have made high contributions to this field. In addition, we have discovered the direction of rapid development that may become a hotspot in the future to attract scholars, which will provide convenience and shortcuts for later research. Over the past 20 years, although the number of articles on HTO surgery has declined in 2011, 2012, 2014, and 2015, it has shown an overall growth trend. This may be due to the rapid increase in the number of patients with knee osteoarthritis with the aging of the population, and the continuous penetration of the concept of “knee protection” has prompted an increasing number of chief surgeons and patients to prefer HTO surgery. Moreover, we predict that the trend in the next 5 years or even 10 years will have an even more significant growth.

No matter in terms of the number of publications, H index, and average citation frequency, the USA is a world leader in the field of HTO. This trend shows that the USA has the highest literature quality and influence and has made the greatest contribution to this field. This is mainly attributed to the USA having the most advanced medical research level and the strongest economic strength around the world. Besides, Germany and South Korea have made outstanding contributions in this field. It is worth mentioning that the number of publications of South Korea surpassed the USA and Germany in 2019. This implies that South Korea has developed rapidly in the HTO field and is a country with great potential (Fig. 2c).

The University of Western Ontario from Canada has published the most articles, with its H index and average cited frequency also ranking second. Obviously, the University of Western Ontario is the biggest contributor in the field of HTO. Furthermore, the second- and third-highest number of publications are from South Korea, another sign that the country is growing rapidly in this field. However, in terms of H index and average citation frequency, one institution in the USA and two institutions in Germany rank top.

As for author contributions, the H index and average citation frequency of the top 10 authors with the highest number of publications were similar, except for Lee YS. Therefore, the number of publications can be considered to be an important criterion for evaluating the contribution of authors. Saito T and Imhoff AB are the leading scholars in the field of HTO according to the number of publications. Paying attention to their research direction and achievements will help us to grasp the latest trends in this field.

*Knee Surgery Sports Traumatology Arthroscopy* has become the most influential journal in the field of HTO with the largest number of articles, the highest H index, and the average cited frequency. Nevertheless, although *Knee* has the second largest number of publications, its H index is low. This may be related to the large number of annual articles published by *Knee*.

The top two funding institutions are all from the USA, which explains one of the reasons why the USA is the largest contributor in this field. Meanwhile, it also suggests that funding plays a vital role in the development of a research field.

Detailed analysis of co-occurrence keyword results will help us grasp the HTO surgery for the future of the research hotspot.

Cluster 1 is “HTO postoperative prognosis and outcome.” HTO is suitable for most young patients due to its advantage of avoiding or delaying total knee arthroplasty [4, 13]. As a surgical method to preserve the knee joint, the postoperative result is a topic of great concern to both doctors and patients [14]. Generally speaking, many kinds of rating scales can be used to measure knee joint function, including KOOS, VAS, and WOMAC score. Multiple studies have confirmed that HTO has good short- and medium-term efficacy [15–17]. However, inevitably, there are still some patients who have to undergo TKA on account of the deterioration of joint degeneration. This involves a controversial issue of widespread concern currently, whether the failure of HTO will affect the efficacy of TKA. A meta-analysis showed that compared with primary TKA, the conversion of HTO to TKA after failure would prolong the operation time, affect the postoperative function, and increase the risk of infection and revision [18]. Undoubtedly, the prognosis of HTO surgery has always been a research direction of interest to scholars, because it is directly related to the life quality of patients.

Cluster 2 is “HTO combined with cartilage restoration techniques.” Cartilage repair technology enriches the treatment of young patients with knee osteoarthritis [19]. Common cartilage restoration techniques include microfracture with abrasion of articular cartilage and autologous chondrocyte transfer. Cartilage restoration techniques improve pain relief after HTO, while HTO support repairs articular cartilage by adjusting the line of force [20]. This collaborative relationship stimulated interest from orthopedic surgeons. Before the birth of cartilage restoration techniques, some scholars believe that the effect of HTO was poor. Many recent studies have shown that HTO combined with cartilage restoration techniques has a good medium-term effect, but the long-term effect remains to be further studied [4, 21, 22]. Technological innovation and long-term efficacy research may be the future development direction in this aspect.

Cluster 3 is “Animal experimental research.” The basic research of HTO is mainly focused on animal experiments. Ziegler et al. concluded that open wedge valgus HTO is a safe surgical method through experiments on sheep [23]. In addition, Franklin et al. found that platelet-rich plasma could not promote bone fracture healing in dogs undergoing HTO [24]. Although this kind of study accounts for less, it is an important part of the HTO research.

Cluster 4 relates to “Study on bone union and plate fixation at osteotomy.” Proper plate fixation is a key factor in the success of HTO operation [25]. Appeared in recent years, many are distinctive of fixed steel plate, such as TomoFix plate, iBalance, Puddu plate, and Contour Lock [26]. Among them, TomoFix plate is widely used. TomoFix plate is designed based on the principle of locking compression plate (LCP), which is strongly fixed by 4 screws at the distal and proximal ends of the osteotomy, so that patients can carry weight and exercise earlier [27]. However, fixation methods need to be tailored because of individual differences (body weight, bone mass, proximal tibial length, tibial shape). Therefore, we speculate that the individual design of the steel plate will become a hot research topic in the future. Nonunion at the end of osteotomy is mainly occurred in medial opening wedge high tibial osteotomy (MOWHTO) but rarely in lateral closing wedge high tibial osteotomy (LCWHTO). Because of the wedge-shaped defect in the medial osteotomy after MOWHTO distraction, whether bone graft is needed or not is still controversial. Most scholars hold the view that bone grafting depends on the spacing of distraction, the angle of correction, whether there is a “hinge” fracture, and the factors that affect the nonunion of the fracture [28–30].

Cluster 5 relates to “Surgical technique research.” At present, there are two main surgical methods for HTO: LCWHTO and MOWHTO. LCWHTO has reliable postoperative stability, allows for early weight-bearing, and does not require bone grafting. Nevertheless, there are also disadvantages such as a high probability of peroneal nerve injury (3.3~11.9%) and unfavorable TKA when the orthotic angle is large [31]. By contrast, MOWHTO has advantages such as the ability to correct coronal and sagittal positions, the minimal possibility of injury to the common peroneal nerve, and simple intraoperative operation. But the nonunion or delayed union of the fracture mentioned above is one of its major defects [32]. OWHTO can more effectively correct the deformity of the force line and meet the needs of young patients by comparing comprehensively these two procedures. Therefore, more attention will perhaps be paid to in the future.

Based on Fig. 4c, it can be noted that most of the red and yellow keywords are distributed in cluster 1, cluster 2, and cluster 5. This indicates that major developments will take place in HTO combined with cartilage restoration techniques, postoperative prognosis and outcome, and surgical technique research in the future.

Although this study provides a comprehensive bibliometric analysis of HTO and the prediction of hot research directions, we believe that there are still the following limitations. First of all, it is restricted by the use of language as only English publications are included. Secondly, we only use WOS database to retrieve data, and several literatures may be omitted. In addition, there is a certain deviation between the results of our bibliometric analysis and the actual results, by reason of the continuous updating of the online database.

## Conclusion

This study reveals the global status and hot direction of HTO. There will be an increasing number of HTO articles published according to the development trend in recent years. The USA has contributed the most in the field of HTO up to now. We speculate that HTO combined with cartilage restoration techniques, postoperative prognosis and outcome, and surgical technique research will may be the future hot directions in HTO field.

## Data Availability

The datasets used and/or analyzed during the current study are not publicly available due to feasibility but are available from the corresponding author on reasonable request.

## Abbreviations

KOA: Knee osteoarthritis
HTO: High tibial osteotomy
WOSCC: The Web of Science Core Collection database
MOWHTO: Medial opening wedge high tibial osteotomy
LCWHTO: Lateral closing wedge high tibial osteotomy
TKA: Total knee arthroplasty
UKA: Unicompartmental knee arthroplasty
